# Acute salivary cortisol response in children with ADHD during psychosocial intervention with and without therapy dogs

**DOI:** 10.3389/fpsyt.2024.1476522

**Published:** 2024-10-24

**Authors:** Sabrina E. B. Schuck, Cassie N. Zeiler, Annamarie Stehli, Lydia A. Steinhoff, Rachel Y. Stokes, Sara E. Jeffrey, Douglas Alan Granger

**Affiliations:** ^1^ Pediatrics, University of California, Irvine, Irvine, CA, United States; ^2^ Pediatrics & Institute for Interdisciplinary Salivary Bioscience, University of California, Irvine, Irvine, CA, United States; ^3^ School of Medicine, Johns Hopkins University, Baltimore, MD, United States

**Keywords:** attention deficit hyperactivity disorder (ADHD), animal assisted interventions, therapy dogs, cortisol (Cor), autism symptomatology, psychosocial skills intervention, school-based intervention

## Abstract

**Introduction:**

Children with Attention Deficit/Hyperactivity Disorder (ADHD) participated in a randomized clinical trial comparing animal-assisted intervention (AAI) to psychosocial treatment as usual (TAU). This brief report describes effects of AAI on acute HPA axis reactivity and regulation. Saliva was collected before, during, and after psychosocial intervention sessions with and without therapy dogs and later assayed for cortisol (ug/dL).

**Methodology:**

Thirty-nine participants (n = 39) with ADHD, aged 7-9 years (79% male) provided saliva at 3 points during 90-minute sessions; (*i*) upon arrival, (*ii*) +20 minutes, and (*iii*) 15 minutes prior to departure, on 3 occasions across an 8-week intervention (weeks 1, 4, and 8). Cortisol slopes calculated within each session were compared across the intervention weeks to determine within subject and between group effect sizes. Spearman’s correlations between baseline individual neurodevelopmental symptoms and in-session acute cortisol responses were also evaluated.

**Results:**

No significant between group differences were observed in cortisol responsiveness at week-1. By week-4, in-session changes in cortisol were evident, with significantly greater decreases in the AAI group (Cohen’s *d* = -.40). This pattern was also observed at week-8, with an even stronger effect-size (*d* = -0.60). Concurrent symptoms of autism were associated with the in-session acute cortisol response. Specifically, higher parent-reported symptom scores were associated with steeper decreases in cortisol across the session at week 1 (*r* = -0.42, *p <*.01) and week-8 (*r* = -0.34 *p* = .05). At week-8 this association was stronger in the AAI group (*r* = -0.53) versus TAU (*r* = -0.25), with Cohen’s *q* = 0.413).

**Discussion:**

AAI may influence acute HPA reactivity and regulation for children with ADHD. Concurrent symptoms of ADHD and autism may be related to individual differences in the nature of the effect. Implications of these findings for AAI as an alternative, or complementary intervention for ADHD are discussed.

**Clinical trial registration:**

ClinicalTrials.gov, identifier NCT05102344.

## 1 Introduction

Despite decades of research aimed to optimize outcomes for children with ADHD, the condition remains a significant public health problem. Pharmacotherapy (e.g., methylphenidate) is the mainstay of traditional medical intervention for ADHD, but side effects (insomnia, anorexia, movement tics) and treatment failures are common (1–3). Of particular concern in the recent years, children with ADHD are often prescribed medications during what are now recognized as critical periods of growth and there is emerging evidence that the dose and frequency of stimulant medicines has varying effects on growth, especially height (4). While the benefits of medication treatments are well-established and oftentimes an integral component of optimal outcomes, it is not surprising that parents and mental health professionals continue to seek complementary and alternative treatments for children with ADHD. Our previous research found AAI to be effective in reducing ADHD symptoms and improving social skills and self-perception (5–7). While we demonstrated efficacy, the underlying mechanism of effect is unknown; a critical knowledge gap needed to increase the acceptability and accessibility of this integrative health care strategy. The present study examines a candidate bio-social mechanism which may influence outcomes of AAI and seeks to better identify individual differences in these biological responses thought to moderate key outcomes.

The suspected mechanisms by which Human Animal Interaction (HAI) is theorized to influence behavior change are diverse. Studies have reported physiological responses to animal interaction indicative of reduced stress as measured by decreased Hypothalamic Pituitary Adrenal axis (HPA) activity as measured by salivary cortisol levels; reduced blood pressure; and decreased Autonomic Nervous System (ANS) activity as measured by increased heart rate variability (HRV) (8). Recent studies have found that AAI (with dogs) lowered salivary cortisol in children (9, 10). One possibility is that these physiological responses reflect a calming-stress reducing effect of AAI and may improve access to curriculum and therapeutic activities (11). Despite these advances there remain several untested alternative rival hypotheses.

The role of individual differences, both biological and psychosocial, and the plausible influences of those differences on response to AAI is a critical area for evaluation. Of interest, a single nucleotide polymorphism (sNP) in the oxytocin receptor gene, *OXTR* rs53576 has long been associated with human social interaction styles (12, 13). This polymorphism, which involves a guanine (G) to adenine (A) substitution, has been extensively studied and those individuals with the A-carrier variant (AA and AG versus GG) may be less sensitive to the social environment (13). A notable finding indicates the quality of children’s interaction with their family pet is moderated by this genotypic difference, in that A-carriers were more engaged with petting their dogs (14). This finding is important as it provides the first evidence for individual genotypic differences that may contribute to differential responses to AAI, particularly for those children who may have social skills deficits.

While long considered a disorder marked by deficits in skills of executive functioning (EF), individuals with ADHD oftentimes are most impaired by social difficulties including oppositionality and a lack of understanding social context. Many will present with co-occurring neurodevelopmental and disruptive behavior disorders, including Oppositional Defiant Disorder (ODD) and Autism Spectrum Disorder (ASD). Specifically, nearly half of individuals with ADHD present with (ODD) (15) and the lifetime prevalence of ADHD in individuals with ASD is estimated at about 40% (16). Of note, a recent review indicates that these disorders have overlapping deficits in skills of executive function (EF) and yet very different psychosocial trajectories (16). Considering the complexity of ADHD, exploration of how these individual differences may moderate response to AAI is indicated. It is reasonable to suspect that differences in response to AAI may be linked to individual differences among children with ADHD.

Deficits in skills of EF, particularly sustained attention and inhibition or self-regulation are associated with differences in activation of and dysregulation of the HPA axis, which is typically measured by cortisol levels. Of note, the secretion of cortisol follows a diurnal cycle and Isaksson et al. (17), reported that children with ADHD, aged 10–17 display lower levels of salivary cortisol in the morning and evening when compared to controls (17). An association of downregulated HPA axis and ADHD fits with theories that regard impairment from ADHD because of under-arousal of the dopaminergic systems (18). Lee, Shin, & Stein (19) studied salivary cortisol response to a stressful stimulus in children with ADHD and found a relationship between increased cortisol levels after the stressor and variability in response time. Taken together these works provide evidence that children with ADHD display higher physiological reactivity to stressors, in line with well-established deficits of self-regulation and inhibition. Studies in typical populations and in children with ASD have linked HAI with acute reductions in cortisol and lowered diurnal patterns in response to AAI (8, 20). It is not known, however, if AAI acts on HPA axis similarly in individuals with low baseline HPA axis activity such as children with ADHD. If so, this reduction could contribute to beneficial outcomes.

In our earlier work, as early as 2 weeks into intervention participants in the AAI group were found to have significant treatment gains compared to the treatment as usual group (TAU) (5). Consistent findings have been reported across different populations of children and diverse animal species (21–23). These findings support proof of the concept that AAI is effective for children with ADHD. None of these studies, however, directly investigated mechanisms of action. Exploration of theoretically indicated mechanisms suspected to elicit benefit from AAI in children with ADHD is indicated. Considering the physiology of ADHD, the hypothetical mechanisms of action in AAI described in other populations may be different in this population. It is not clear that AAI elicits the same physiological responses in children with ADHD as it does in other populations. Exploration into the potential role of HPA axis reactivity and other individual differences on AAI outcomes may inform both practices and policies.

## 2 Method

### 2.1 Study design

This study was approved by the local university Institutional Review Board (IRB) as well as the Institutional Animal Care and Use Committee (IACUC). This study employed an exploratory parallel group randomized controlled trial study design and utilized a *multi-method and multi-source* assessment protocol. Parents or legal guardians of all participants provided signed consent for their minor to participate and all child participants provided written or verbal assent. After completing consent and meeting eligibility criteria, participants were randomly assigned to one of two intervention conditions, group psychosocial skills training treatment as usual (TAU; n = 19), or (2) the same group treatment assisted by therapy dog/handler dyads or animal assisted intervention (AAI; n = 20).

### 2.2 Participants

Thirty-nine (39) children, ages 7 to 9 (79% male) were randomly allocated to intervention conditions (TAU or AAI) and completed their respective group intervention across six (6) cohorts over two (2) years. Eligibility was determined utilizing a multi-gate screening procedure described in our earlier work including a screening for history of stimulant treatment, animal abuse, fear of dogs, and allergy to dogs. All participants met research criteria for combined-type attention deficit/hyperactivity disorder (ADHD). Individuals meeting research criteria for autism spectrum disorder (ASD), as measured using the Kaufman-Schedule for Affective Disorders and Schizophrenia for School-Age Children (K-SADS) were excluded (24). Additionally, participants had at least an estimated full-scale IQ of 80 as determined by the Wechsler Abbreviated Scale of Intelligence (25). Finally, all participants were either medication naive or had experienced at least a 6-week wash out of any stimulant medications prior to consent and screening.

### 2.3 Therapy dog/handler dyads

Two (2) volunteer therapy dog/handler dyads were selected to participate over the course of the study after completing a screening process and interview described elsewhere (26). Both dyads were registered through Pet Partners, a volunteer therapy animal organization. Pet Partners requires that all dog/handler dyads participate in a rigorous initial qualifying process and are regularly evaluated using a rigorous set of standards for safety, behavior, and conduct. Standards for humane involvement of animals and their welfare during AAI set forth in the International Association of Human Animal Interaction Organization’s white paper (27) were also adhered to throughout the study to best ensure the safety and general welfare of the dogs. The study was assisted by a 5-year-old, male Golden Retriever, and his volunteer female hander (aged 60) and a 3-year-old, female English Cream Retriever, and her volunteer female handler (aged 59). Handlers participated throughout the AAI to ensure the safety and comfort of the therapy dogs. To prevent fatigue, the duration of the dog involvement was limited to 90-minutes of the two-hour sessions.

### 2.4 Intervention models

Conventional treatments for children with ADHD include stimulant medications, behavioral parent education, and psychosocial skills training interventions (SST). A manualized social skills intervention with and without the assistance of volunteer therapy dog/handler dyads was developed in our prior work was adapted for this pilot study (5). The Positive Assertive Cooperative Kids (PACK) model targets the reduction of symptoms of inattention, hyperactivity, and oppositional behavior and the development of self-awareness, self-regulation, and pro-social behavior (6). The PACK model can be implemented with (AAI) or without (TAU) the assistance of volunteer therapy dog dyads. Participants in the TAU condition followed the same protocol as the AAI, but activities utilized realistic puppets in lieu of therapy dogs. In the evidence-based model, six children attended group social skills training sessions twice weekly over a 10-week intervention with or without three accompanying therapy dog dyads. For the aims of the current pilot study, the intervention was reduced to be delivered once weekly across an 8-week intervention. Furthermore, due to COVID-19 global pandemic and government issued safety protocols, recruitment and participant attendance was compromised for this pilot. As such modifications were made to the original PACK model to allow for four to five children per group and one or two volunteer dog/handler dyads accompanying one moderator and two behavior counselors.

Participants in each group were taught the same social skills (i.e., assertion, accepting, ignoring, etc.) complemented with lessons on humane treatment, animal welfare, and safe interactions with dogs. In the AAI group, participants were also instructed on how to teach and deliver basic commands for dogs (i.e., sit, down, stay), while the TAU group practiced teaching skills utilizing telling stories or sharing about themselves with their peers. Participation in the lessons was supported by a token economy system delivered by the two behavior counselors. Treatment fidelity across sessions and weeks for both conditions was supported by utilizing a manualized intervention checklist observation measure and post-session intervention team debriefing each week.

### 2.5 *In-vivo* saliva collection

To explore HPA axis activity, an indicator of an individuals’ stress response, saliva was collected from all participants utilizing Salimetrics^®^ SalviaBio Oral Swab (SOS method). All participants provided saliva samples at each of three time points during two-hour after-school intervention sessions, (*i*) upon arrival at approximately 4:00 p.m., (*ii*) +20 minutes, and (*iii*) 15 minutes prior to the end of the lesson at approximately 5:20 p.m. Of note, parents were instructed to ensure that their children refrain from eating at least 30-minutes and preferably one hour prior to arrival to the sessions. During collection procedures, participants were directed to remain seated in a chair for two minutes with the oral swab inserted in their mouth. After the two-minute period, participants were directed to spit the swab into a uniquely labeled vial corresponding with their participant identification number. Each vial was immediately placed in frozen storage at -20° Celsius. Time stamps were recorded for the times when: a) the swab entered the child’s mouth, b) the child spat the swab into the vial, and c) the vial was put into placed into frozen storage. This collection and storage procedure was repeated on three total occasions across an 8-week intervention (at weeks-1, 4, and 8). Saliva samples were then tested for cortisol using a commercially available immunoassay specifically designed for use with saliva at the Salimetrics Technology and Development Center (Carlsbad, CA). The assay had a lower limit of sensitivity of.007 ug/dl and range up to 3.0 ug/dL with average inter and intra-assay coefficients of variation less than 15 and 5%, respectively.

## 3 Analysis

### 3.1 Salivary cortisol

Due to design factors such as reduced subgroup sample size, non-normally distributed measures, repeated measurements and the potential for relatively high variability, a combination of non-parametric statistical analyses and effect sizes were employed. This allowed both statistical evaluation of the intervention and exploration of clinical significance as well as effect modifiers. Acute HPA axis response to intervention conditions during sessions was measured by calculating three slopes for change in Cort (ug/dL) among the three in-session time points for all participants (1); from arrival to +20 minutes (*i* to *ii*) (2), from +20 minutes to 15 minutes prior to lesson end (*ii* to *iii*) and (3) from arrival to 15 minutes prior to lesson end (*i* to *iii*). These calculations were repeated on three occasions across the intervention period, at weeks-1, 4, and 8. Concentration values (ug/dL) at each timepoint were also examined. Average slopes and concentrations were compared within-subject (over time) and between groups utilizing repeated measures analyses (Wilcoxon sign rank tests for paired comparisons) and effect size calculations (28).

### 3.2 Individual differences

The individual neurodevelopmental symptoms of ADHD, Oppositional Defiant Disorder, Autism Spectrum Disorder, reading skills and intellectual skills were measured at baseline utilizing a battery of assessment tools Attention Deficit Hyperactivity Disorder Rating Scale (29), Autism Syndrome Rating Scale (30), Test of Word Reading Efficiency (31), and the Wechsler Abbreviated Scale of Intelligence (25). The relationship between each of these measures and acute sCort in-session response were explored utilizing Spearman’s *r* correlation coefficients within each group (AAI and TAU). Comparison of correlation coefficients between groups were performed using effect sizes (28).

## 4 Results

**Figure 1:**
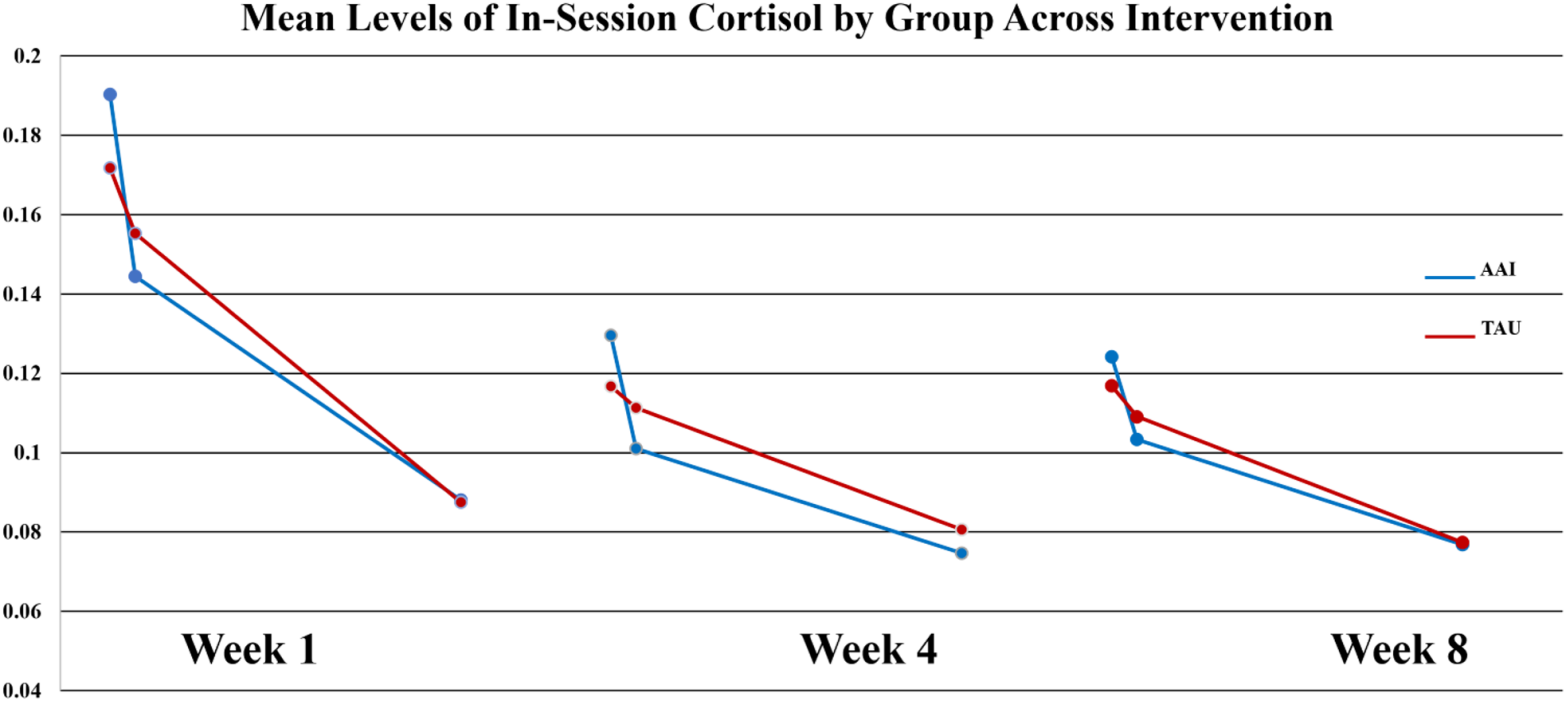
Mean levels of in-session cortisol by group across intervention.

**Figure 2:**
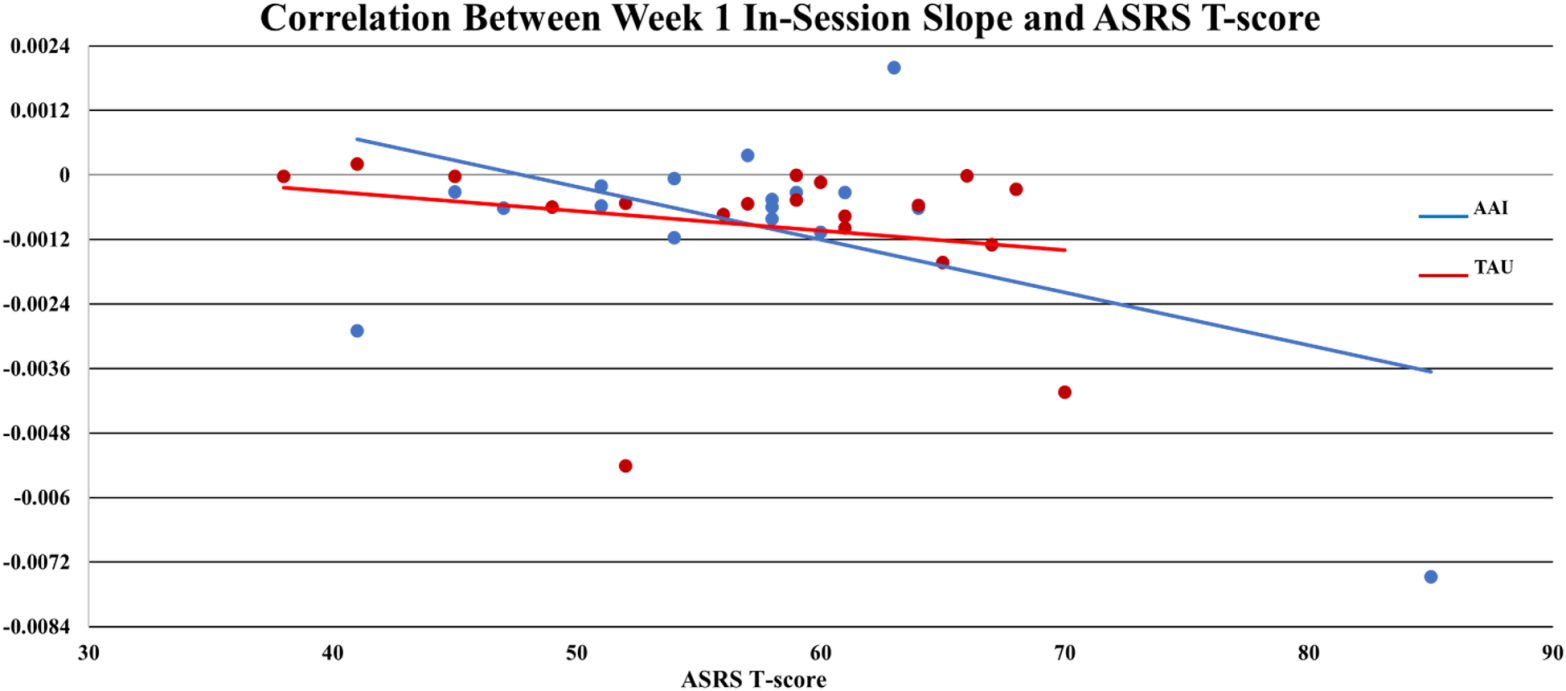
Correlation between week 1 in-session slope and ASRS t-score.

**Figure 3:**
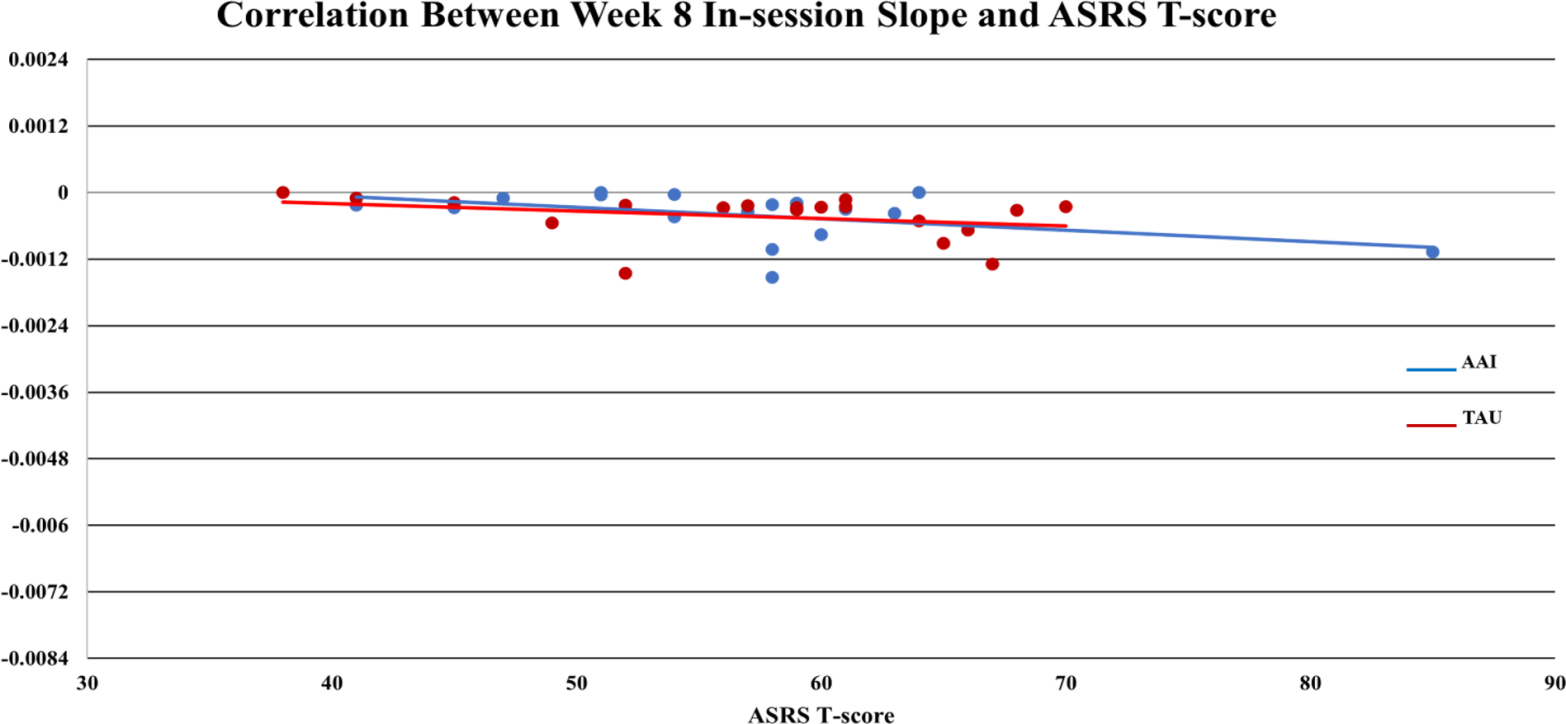
Correlation between week 8 in-session slope and ASRS t-score.

At week-1, there were no significant group differences in any of the three sCort slopes measured across the 90-minute lesson session (p = .727, p = .834, p = .646). By week-4, group differences in the first cortisol slope upon arrival (ΔCort arrival to +20 minutes) were revealed, with the dog group demonstrating a steeper decrease in cortisol on average (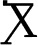
_AAI_ = -0.0014, 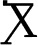
_TAU_ = -0.0003). This difference produced a moderate effect size (*d* = .547). and approached statistical significance on non-parametric tests (Wilcoxon *S* p =. 0.0562. At week-8 this trend was still present (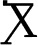
_AAI_ = -0.0010, 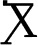
_TAU_ = - 0.0004), but the effect was smaller (*d* = -.386, *p* = 0.365). Of note, for all participants mean values of sCort were relatively high at arrival on week-1 (0.1813 µg/dL) with a greater magnitude of reduction within 20-minutes for the AAI group. The effect size for this reduction was medium (*d* = .468) though not statistically significant (*p* = .376). This trend was consistent over time (See **Figure 1**). At week-1, for all participants, symptoms of autism, as measured by parent reported ASRS total *t*-score at enrollment, were significantly correlated with the overall in-session slope (arrival to 15 minutes prior to departure) (*r* = -0.3736, *p* = .025). Of note, the arrival slope at week-1 (arrival to +20 minutes) was moderately correlated with the ASRS (*r* = - 0.4756, *p* = .003) and this correlation seems to be contributing to the overall in-session correlation. Specifically, higher *t*-scores on the ASRS were associated with steeper decreases in sCort across the session (See **Figure 2**). At week-8, the overall in-session slope (arrival to 15 minutes prior to departure) correlation remained significant for both intervention groups (*r* = -0.3685, *p* = .035). Moreover, in week-8, the relationship was larger in the AAI group (*r* = -0.508) versus TAU (*r* = - 0.2144) yielding a medium effect size (Cohen’s *q* = 0.376) (See **Figure 3**). No other individual differences in screening measures were revealed (i.e., ADHD or ODD symptom severity, IQ, gender, age, etc.)

## 5 Discussion

Results suggest this AAI, adapted from the manualized intervention protocol employed in our earlier work, lowers in-session HPA axis activity in children with ADHD. This response provides evidence that AAI may play a role in improving treatment outcomes of more traditional psychosocial interventions. Additionally, these findings suggest that even in reduced ‘doses’ interacting with volunteer therapy dog results in measurable physiological responses associated with reduced stress.

### 5.1 The role of individual differences

The role individual differences may play in the response to psychosocial interventions and to AAI have been discussed in the literature (14, 32). Of interest, in our findings, is the role of co-occurring symptoms of autism in physiological responses to AAI among children with ADHD who did not meet diagnostic criteria for ASD. Specifically, we found a moderate correlation in the initial reduction in cortisol during the first 20 minutes of the intervention and symptoms of autism, suggesting that those individuals with more social impairment may particularly benefit from the accompaniment of a therapy dog during evidence-based psychosocial intervention. It has been shown that animal assisted activities (AAA) are effective in improving social functioning in young children with ASD (33). Considering the present results, and the well-established overlap of symptomatology in children with ADHD and ASD, we suspect there may be a similar mechanism of action underlying favorable response to AAI.

### 5.2 Biological sensitivity to stress and the social context

Considering the complexity of ADHD presentation, it is reasonable to surmise that children with ADHD may be more, or less, biologically sensitive to the context in which therapeutic interventions are delivered. We suspect that those who are the most biologically sensitive to negative experiences and less sensitive to social feedback are also the ones who are the most likely to benefit from an AAI enriched treatment. A closer examination of biological responses to AAI for children with ADHD could provide information about what profiles of children are most likely to benefit from AAI. It may be that some children with ADHD do not present with the same degree of biological sensitivity to the context in which psychosocial interventions are delivered but that others are particularly sensitive and stand to benefit more from a treatment enriched by AAI.

This work contributes to a growing body of evidence supporting the efficacy of involving animals in activities and interventions for special populations of children. Biometric data collected from the present study contributes to field of HAI research by enhancing our understanding of the biosocial mechanisms by which AAI can improve outcomes for children with ADHD as well as yields important findings regarding individual differences in response to AAI. This work also demonstrates that, even in lower doses than previously implemented, AAI still yields meaningful clinical improvement. Additionally, this study establishes the feasibility of conducting salivary science methods during AAI for this population.

## 6 Future directions & limitations

There is limited understanding of the socio-emotional and physiological mechanisms by which interaction with animals has therapeutic benefit for this population; specifically, when and for whom AAI with therapy dogs may be most effective. This pilot work indicates the role of stress response to AAI may play an important role in understanding how this population benefits from AAI. Without delineation of the mechanisms of AAI in this population, the development of rigorous empirical studies is hampered. This work suggests cortisol, a biomarker for HPA axis activity, is a viable candidate mechanism involved in response to AAI. Next steps include the need to explore the relationship of this biomarker and social outcomes of self-regulation and self-awareness, key components of executive function. Future studies, including larger trials including participants with common comorbid neurodevelopmental and behavioral disorders, to examine the potential role of this biomarker in AAI are warranted to better understand the generalizability of this finding. Specifically, results regarding the role of individual differences in response to AAI, especially a more in-depth exploration of complex symptom presentation around social competency is indicated.

The sample size of this exploratory study limits the generalizability of this work. Of note, while designed as an exploratory pilot, with a targeted sample size of 45-48, the intervention protocol was delivered during the global COVID-19 pandemic which compromised enrollment and attrition. Despite the limitations of the study,the results suggest, a further examination of other biological and psychosocial factors related to stress response, particularly ANS reactivity may contribute to our understanding of which other mechanisms may contribute to AAI response. Candidate biomarkers (salivary alpha-amylase and Heart Rate Variability) were also collected in this trial and will be examined. Additionally, parent ratings of prosocial behaviors (social skills) and the quality of observed child-dog interaction during AAI sessions was also collected and will be examined.

## Data Availability

The raw data supporting the conclusions of this article will be made available by the authors, without undue reservation.
